# Robustness and Accuracy in Sea Urchin Developmental Gene Regulatory Networks

**DOI:** 10.3389/fgene.2016.00016

**Published:** 2016-02-15

**Authors:** Smadar Ben-Tabou de-Leon

**Affiliations:** The Department of Marine Biology, The University of HaifaHaifa, Israel

**Keywords:** developmental gene regulatory network, development and evolution, compound network motifs, sea urchins, Wnt signaling pathway, BMP signaling, Delta-Notch signaling

## Abstract

Developmental gene regulatory networks robustly control the timely activation of regulatory and differentiation genes. The structure of these networks underlies their capacity to buffer intrinsic and extrinsic noise and maintain embryonic morphology. Here I illustrate how the use of specific architectures by the sea urchin developmental regulatory networks enables the robust control of cell fate decisions. The Wnt-βcatenin signaling pathway patterns the primary embryonic axis while the BMP signaling pathway patterns the secondary embryonic axis in the sea urchin embryo and across bilateria. Interestingly, in the sea urchin in both cases, the signaling pathway that defines the axis controls directly the expression of a set of downstream regulatory genes. I propose that this direct activation of a set of regulatory genes enables a uniform regulatory response and a clear cut cell fate decision in the endoderm and in the dorsal ectoderm. The specification of the mesodermal pigment cell lineage is activated by Delta signaling that initiates a triple positive feedback loop that locks down the pigment specification state. I propose that the use of compound positive feedback circuitry provides the endodermal cells enough time to turn off mesodermal genes and ensures correct mesoderm vs. endoderm fate decision. Thus, I argue that understanding the control properties of repeatedly used regulatory architectures illuminates their role in embryogenesis and provides possible explanations to their resistance to evolutionary change.

## Introduction

Robustness, the perseverance of phenotype through genetic and environmental changes (de Visser et al., 2003), is a prominent property of embryo development. Thus, embryos can maintain their morphologies through a wide range of temperatures and pH (Runcie et al., 2012; Pespeni et al., 2013; Kuntz and Eisen, 2014) and within substantial genetic variation (Garfield et al., 2013). This robustness of the developmental program relays on various levels of molecular control, among them, transcription factor binding to the DNA, enhancer structure and the architecture of developmental gene regulatory networks (reviewed in de Visser et al., 2003; Kitano, 2007; Payne and Wagner, 2015). Here I describe the repeated use of specific network architectures in the sea urchin developmental gene regulatory networks, and illustrate how they contribute to robust cell fate decision.

The current model of the sea urchin developmental regulatory networks encompasses all the embryonic territories up to gastrulation and is one of the most elaborate of its kind (Saudemont et al., 2010; Peter and Davidson, 2011; Materna and Davidson, 2012; Ben-Tabou de-Leon et al., 2013). A major strength of this network model is the extensive *cis*-regulatory analyses conducted for many nodes (e.g., Nam et al., 2007; Ben-Tabou de Leon and Davidson, 2010; Ransick and Davidson, 2012). Thus, the direct connectivity of this network is highly reliable and can provide a systems level view of how network architecture contributes to the precise control of embryonic axes formation and germ layer specification.

Within the sea urchin regulatory network, specific network architectures are repeatedly used to control various patterning events at different embryonic territories (Ben-Tabou de-Leon and Davidson, 2006; Peter and Davidson, 2009). These network architectures are composed of multiple interconnected common network motifs: switches, feedforward and feedback loops (Ben-Tabou de-Leon and Davidson, 2006; Peter and Davidson, 2009). The concept of “common network motifs” originated more than a decade ago by Alon and colleagues that identified typical three-node network circuitries overrepresented in bacterial transcriptional regulatory networks (Shen-Orr et al., 2002). Since then, similar and other network motifs were identified in other biological systems and their intensive study illuminates the relationship between motif structure and its control function (Hornung and Barkai, 2008; Shoval and Alon, 2010). Here I illustrate how compound interconnected network motifs are used by the sea urchin developmental gene regulatory networks and propose that their control properties are utilized to ensure robustness and accuracy of cell fate decisions.

## Wnt-βcatenin regulation of primary axis formation and endoderm specification

**Figure 1:**
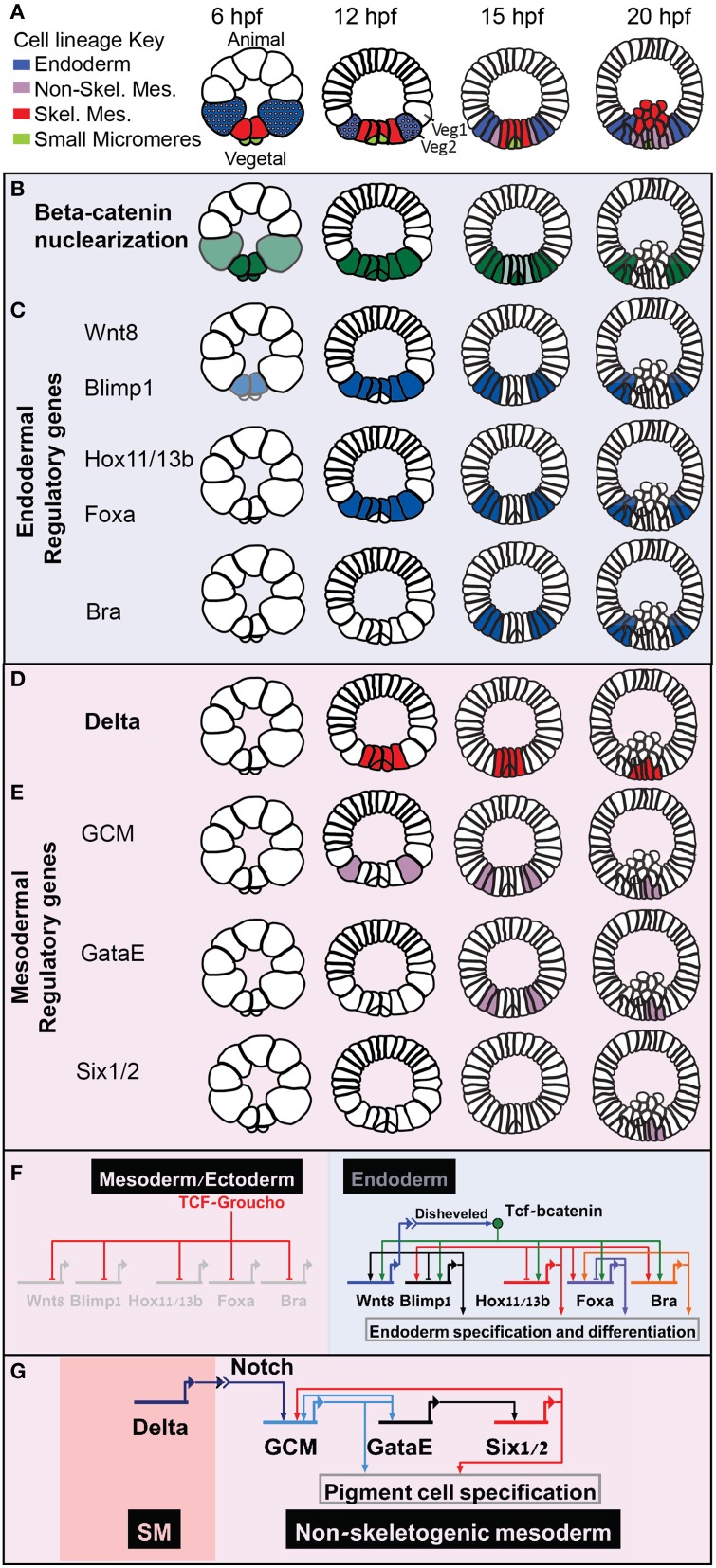
**Sea urchin embryonic development and endoderm specification**. Developmental time is described in hours post fertilization according the developmental rate of the purple sea urchin, *S. purpuratus*. **(A)** Sea urchin endomesoderm cell lineage diagram. Color key is described in the figure. **(B)** βcatenin nuclearization pattern, dark green indicates high concentration, light green low. **(C)** Spatio-temporal expression profiles of endodermal control genes. **(D)** Partial endodermal GRN model depicting Tcf/βcatenin-Tcf/Groucho switch and regulatory interactions within the endodermal genes. **(E)** Spatio-temporal expression of the Delta ligand. **(F)** spatio-temporal expression of non-skeletogenic mesodermal genes. **(G)** GRN model of the triple positive feedback loop that Delta reception activates in the non-skeletogenic cells.

Extensive research had shown the extreme conservation of the role of the Wnt-βcatenin signaling pathway in primary axis formation and endoderm specification across metazoan (Petersen and Reddien, 2009). The model of the sea urchin developmental regulatory networks reveal how Wnt-βcatenin spatial information is transformed into specific cell fate decisions. The primary axis in the sea urchin embryo, the animal-vegetal axis, is initiated by nuclear localization of βcatenin in all the cells of the vegetal half of the embryo [Figure 1A, endomesodermal lineages, B, βcatenin nuclearization pattern (Logan et al., 1999)]. When βcatenin enters the nucleus it forms an activating complex with the transcription factor Tcf that otherwise forms a repressor complex with Groucho. The βcatenin-Tcf switch initiates the specification of both mesoderm and endoderm in the vegetal half of the sea urchin embryo (Figures 1A–E).

βcatenin-Tcf switch directly activates the expression of a set of endodermal regulatory genes, *hox11/13, blimp1, foxa*, and *bra*, in a staggered manner [Figure 1C (Cui et al., 2014)]. That is, the expression of each of these gene is turned on at a different time, but their spatial expression overlap, at least at the earlier stages of their expression (Minokawa et al., 2005; Livi and Davidson, 2006; Peter and Davidson, 2010, 2011). Each of these genes has functional Tcf sites in its enhancers, indicating direct control of Wnt signaling through βcatenin /Groucho-Tcf switch (Figure 1F, Minokawa et al., 2005; Smith et al., 2007, 2008; Ben-Tabou de Leon and Davidson, 2010).

At Mesenchyme blastula stage, βcatenin clears from the mesodermal nuclei, first from the skeletogenic lineage and then from the non-skeletogenic mesoderm [Figure 1B (Logan et al., 1999)]. When βcatenin is cleared from the mesodermal nuclei the Tcf sites on the enhancers of the endodermal genes control their clearance from the mesoderm territories through Tcf-Groucho mediated repression (Ben-Tabou de Leon and Davidson, 2010) and thus regulate the endoderm—mesoderm cell fate decision (Figure 1F, Peter and Davidson, 2011). Apparently, βcatenin-Tcf acts as a permissive switch and restricts the expression of these genes spatially, while their differential activation time is defined by their specific activators (Figure 1F). I suggest that this mode of regulatory circuitry decouples the spatial from the temporal regulation and promotes a uniform spatial response of all the endodermal genes. Thus, βcatenin-Tcf/Groucho-Tcf switch ensures that the endodermal genes will be cleared from the mesodermal domain at the right developmental stage and guarantees a clear-cut cell fate decision.

## Delta-notch activation of a triple positive feedback circuit and mesoderm cell fate specification

The Delta-Notch signaling pathway is highly conserved in metazoan and controls glial vs. neural differentiation (Gaiano and Fishell, 2002). Early in sea urchin embryogenesis, the gene that encodes the ligand Delta is activated indirectly by the βcatenin-Tcf input in the skeletogenic mesoderm (Figure 1D, Oliveri et al., 2008). The reception of Delta in the neighboring tier of cells, Veg2, activates the gene that encodes the transcription factor *glial cells missing* [GCM, Figures 1E,G (Ransick and Davidson, 2006; Croce and McClay, 2010)]. GCM then establishes a triple positive feedback loop by directly activating the expression of the transcription factor GataE, that activates the expression of the transcription factor Six1/2, that feeds back to activate GCM expression (Figures 1E,G, Ransick and Davidson, 2012). GCM-GataE-Six1/2 triple positive feedback loop maintains the expression of these three genes in the pigment cell lineage after Delta signal stops being received in these cells (20 hpf in *S. purpuratus*, Figures 1D,E,G).

The tier of cells where GCM is first activated, Veg2, give rise to both endoderm and non-skeletogenic mesoderm lineages (Figure 1A, 12 hpf). When Veg2 cells divide, only the future pigment cells remain in direct contact with the Delta secreting SM cells, while the future endodermal cells lose this contact and therefore lose the Delta signal (Figure 1A, 15 hpf). Hence, in the endodermal cells the Delta signal is not received long enough to establish the triple positive feedback loop so GCM expression turns off there (Figure 1E, 15 and 20 hpf, Ransick and Davidson, 2006, 2012; Croce and McClay, 2010). The transient Delta signal is practically filtered in the endodermal cells by the mesodermal positive feedback loop to allow correct endodermal fate decision.

Previous theoretical studies of three component circuits show that feedback circuitry is more efficient than other architectures in buffering noise in the inducing signal while keeping high responsivity to the level of the signal (Hornung and Barkai, 2008). According to these studies, noise reduction in positive feedback circuits results from effectively slowing the response dynamics and allowing for better averaging of the induction signal over time. Additionally, mathematical modeling of the kinetics of positive feedback loops shows that compound positive feedback circuitry is less responsive than single positive feedback loop to low levels of activating signals (Ben-Tabou de-Leon, 2010). These studies suggests that compound positive feedback circuitry filters better low and transient signals compared to single positive feedback loops and thus are a more reliable mechanism for regulatory state lock down. This could be the reason for the common use of compound positive feedback circuits by developmental networks instead of single gene positive feedback loop.

## TGFb pathways control of secondary axis and ectoderm specification

**Figure 2:**
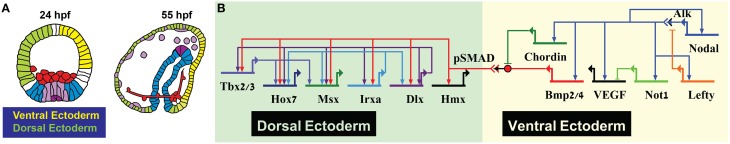
**Sea urchin dorsal-ventral patterning. (A)** Sea urchin lineage diagram showing ventral (yellow) and dorsal (light green) ectoderm. **(B)** Partial model on Dosrsal-Ventral patterning in the sea urchin depicting key regulatory processes in the ectoderm.

The gene regulatory networks that pattern the secondary embryonic axis, the dorsal-ventral axis of the sea urchin embryo, use similar circuit architectures to those discussed above. Nodal signaling directly activates the ventral ectoderm regulatory genes that then interact with each other to form subdomains within the ventral ectoderm [Figure 2 (Saudemont et al., 2010; Li et al., 2014)]. Two of Nodal targets at the ventral ectoderm are the ligand BMP2/4 and its inhibitor Chordin. Chordin inhibits BMP reception at the ventral side so the mediator of BMP signaling, the transcription factor SMAD1/5/8, is phosphorylated and activates transcription only in the dorsal side of the embryo (Figure 2B, Saudemont et al., 2010; Ben-Tabou de-Leon et al., 2013). BMP operates in a feed-forward structure, directly activating the expression of dorsal transcription factors that then regulate one another forming compound positive feedback loop (Figure 2B, Ben-Tabou de-Leon et al., 2013). Thus, BMP provides a temporal cue that uniformly boosts the expression of the aboral transcription factors at the exact time when the first genes that specify the neighboring territory, the ciliated band, are turning on (Ben-Tabou de-Leon et al., 2013).

## Conclusions: precise and highly conserved control of expression dynamics

As we gain more information on the structure and function of gene regulatory networks we can start asking why are specific architectures used more than others and why are they so deeply conserved? A recent paper revealed remarkable conservation of regulatory gene expression dynamics between two sea urchin species after 40 million years of independent evolution (Gildor and Ben-Tabou de-Leon, 2015). The use of direct activation by signaling pathways and compound positive feedback circuitry described above could underlie this strong conservation of expression dynamics and the observed robustness within genotypic variance and different environmental conditions.

Direct activation by a signaling pathway might be a general strategy used by developmental gene regulatory networks to guarantees a uniform timely response of a set of key regulatory genes. This strategy could also explain the deep conservation of the role of Wnt and BMP pathways in primary embryonic axes specification. If the activation of the downstream gene regulatory network was in a cascade of regulatory interactions, there were only a few regulatory changes required to replace Wnt or BMP with alternative signaling input. It is much less likely to replace Wnt or BMP signaling when they activate the entire set of genes that define the endoderm or dorsal ectoderm specification, respectively. Thus, the direct activation of large sets of regulatory genes by signaling pathways might be important for clear cut cell fate decision on one hand, and on the other hand imposes a strong constraint on the use of these signaling pathways in developing embryos.

Similar argument could explain the extreme conservation of well-studied compound positive feedback circuits. Specifically, the compound positive feedback circuit that controls the lock down of endoderm cell fate specification was conserved across 500 years of echinoderm evolution (Hinman et al., 2003); the compound positive feedback circuit that controls heart development is conserved between human and fly (Olson, 2006). It seems that any regulatory change within these critical control circuits must have reduced the circuit precision and therefore had been selected against. Thus, understanding the control properties of repeatedly used regulatory architectures illuminates their function in developing embryos and provides possible explanation to their resistance to evolutionary change.

## Author contributions

The author confirms being the sole contributor of this work and approved it for publication.

## Funding

This work was supported by the Marie Curie Carrier Integration Grant FP7-PEOPLE-2012-CIG, grant number 321758.

### Conflict of interest statement

The author declares that the research was conducted in the absence of any commercial or financial relationships that could be construed as a potential conflict of interest.

## References

[B1] Ben-Tabou de-Leon S. (2010). Perturbation analysis analyzed - mathematical modeling of intact and perturbed gene regulatory circuits for animal development. Dev. Biol. 344, 1110–1118. 10.1016/j.ydbio.2010.06.020 20599898 PMC2920143

[B2] Ben-Tabou de Leon S. Davidson E. H. (2010). Information processing at the foxa node of the sea urchin endomesoderm specification network. Proc. Natl. Acad. Sci. U.S.A. 107, 10103–10108. 10.1073/pnas.1004824107 20479235 PMC2890477

[B3] Ben-Tabou de-Leon S. Davidson E. H. (2006). Deciphering the underlying mechanism of specification and differentiation: the sea urchin gene regulatory network. Sci. STKE 2006:pe47. 10.1126/stke.3612006pe47 17106076

[B4] Ben-Tabou de-Leon S. Su Y. H. Lin K. T. Li E. Davidson E. H. (2013). Gene regulatory control in the sea urchin aboral ectoderm: spatial initiation, signaling inputs, and cell fate lockdown. Dev. Biol. 374, 245–254. 10.1016/j.ydbio.2012.11.013 23211652 PMC3548969

[B5] Croce J. C. McClay D. R. (2010). Dynamics of Delta/Notch signaling on endomesoderm segregation in the sea urchin embryo. Development 137, 83–91. 10.1242/dev.044149 20023163 PMC2796929

[B6] Cui M. Siriwon N. Li E. Davidson E. H. Peter I. S. (2014). Specific functions of the Wnt signaling system in gene regulatory networks throughout the early sea urchin embryo. Proc. Natl. Acad. Sci. U.S.A. 111, E5029–E5038. 10.1073/pnas.1419141111 25385617 PMC4250154

[B7] de Visser J. A. Hermisson J. Wagner G. P. Ancel Meyers L. Bagheri-Chaichian H. Blanchard J. L.. (2003). Perspective: evolution and detection of genetic robustness. Evolution 57, 1959–1972. 10.1111/j.0014-3820.2003.tb00377.x 14575319

[B8] Gaiano N. Fishell G. (2002). The role of notch in promoting glial and neural stem cell fates. Annu. Rev. Neurosci. 25, 471–490. 10.1146/annurev.neuro.25.030702.130823 12052917

[B9] Garfield D. A. Runcie D. E. Babbitt C. C. Haygood R. Nielsen W. J. Wray G. A. (2013). The impact of gene expression variation on the robustness and evolvability of a developmental gene regulatory network. PLoS Biol. 11:e1001696. 10.1371/journal.pbio.1001696 24204211 PMC3812118

[B10] Gildor T. Ben-Tabou de-Leon S. (2015). Comparative study of regulatory circuits in two sea urchin species reveals tight control of timing and high conservation of expression dynamics. PLoS Genet. 11:e1005435. 10.1371/journal.pgen.1005435 26230518 PMC4521883

[B11] Hinman V. F. Nguyen A. T. Cameron R. A. Davidson E. H. (2003). Developmental gene regulatory network architecture across 500 million years of echinoderm evolution. Proc. Natl. Acad. Sci. U.S.A. 100, 13356–13361. 10.1073/pnas.2235868100 14595011 PMC263818

[B12] Hornung G. Barkai N. (2008). Noise propagation and signaling sensitivity in biological networks: a role for positive feedback. PLoS Comput. Biol. 4:e8. 10.1371/journal.pcbi.0040008 18179281 PMC2174979

[B13] Kitano H. (2007). Towards a theory of biological robustness. Mol. Syst. Biol. 3:137. 10.1038/msb4100179 17882156 PMC2013924

[B14] Kuntz S. G. Eisen M. B. (2014). Drosophila embryogenesis scales uniformly across temperature in developmentally diverse species. PLoS Genet. 10:e1004293. 10.1371/journal.pgen.1004293 24762628 PMC3998915

[B15] Li E. Cui M. Peter I. S. Davidson E. H. (2014). Encoding regulatory state boundaries in the pregastrular oral ectoderm of the sea urchin embryo. Proc. Natl. Acad. Sci. U.S.A. 111, E906–E913. 10.1073/pnas.1323105111 24556994 PMC3956148

[B16] Livi C. B. Davidson E. H. (2006). Expression and function of blimp1/krox, an alternatively transcribed regulatory gene of the sea urchin endomesoderm network. Dev. Biol. 293, 513–525. 10.1016/j.ydbio.2006.02.021 16581059

[B17] Logan C. Y. Miller J. R. Ferkowicz M. J. McClay D. R. (1999). Nuclear beta-catenin is required to specify vegetal cell fates in the sea urchin embryo. Development 126, 345–357. 9847248 10.1242/dev.126.2.345

[B18] Materna S. C. Davidson E. H. (2012). A comprehensive analysis of Delta signaling in pre-gastrular sea urchin embryos. Dev. Biol. 364, 77–87. 10.1016/j.ydbio.2012.01.017 22306924 PMC3294105

[B19] Minokawa T. Wikramanayake A. H. Davidson E. H. (2005). cis-Regulatory inputs of the wnt8 gene in the sea urchin endomesoderm network. Dev. Biol. 288, 545–558. 10.1016/j.ydbio.2005.09.047 16289024

[B20] Nam J. Su Y. H. Lee P. Y. Robertson A. J. Coffman J. A. Davidson E. H. (2007). Cis-regulatory control of the nodal gene, initiator of the sea urchin oral ectoderm gene network. Dev. Biol. 306, 860–869. 10.1016/j.ydbio.2007.03.033 17451671 PMC2063469

[B21] Oliveri P. Tu Q. Davidson E. H. (2008). Global regulatory logic for specification of an embryonic cell lineage. Proc. Natl. Acad. Sci. U.S.A. 105, 5955–5962. 10.1073/pnas.0711220105 18413610 PMC2329687

[B22] Olson E. N. (2006). Gene regulatory networks in the evolution and development of the heart. Science 313, 1922–1927. 10.1126/science.1132292 17008524 PMC4459601

[B23] Payne J. L. Wagner A. (2015). Mechanisms of mutational robustness in transcriptional regulation. Front. Genet. 6:322. 10.3389/fgene.2015.00322 26579194 PMC4621482

[B24] Pespeni M. H. Chan F. Menge B. A. Palumbi S. R. (2013). Signs of adaptation to local pH conditions across an environmental mosaic in the California Current Ecosystem. Integr. Comp. Biol. 53, 857–870. 10.1093/icb/ict094 23980118

[B25] Peter I. Davidson E. H. (2010). Genomic programs for endoderm specification in sea urchin embryos. Dev. Biol. 344:469 10.1016/j.ydbio.2010.05.225 PMC3981691 19895806

[B26] Peter I. S. Davidson E. H. (2009). Modularity and design principles in the sea urchin embryo gene regulatory network. FEBS Lett. 583, 3948–3958. 10.1016/j.febslet.2009.11.060 19932099 PMC2810318

[B27] Peter I. S. Davidson E. H. (2011). A gene regulatory network controlling the embryonic specification of endoderm. Nature 474, 635–639. 10.1038/nature10100 21623371 PMC3976212

[B28] Petersen C. P. Reddien P. W. (2009). Wnt signaling and the polarity of the primary body axis. Cell 139, 1056–1068. 10.1016/j.cell.2009.11.035 20005801

[B29] Ransick A. Davidson E. H. (2006). cis-regulatory processing of Notch signaling input to the sea urchin glial cells missing gene during mesoderm specification. Dev. Biol. 297, 587–602. 10.1016/j.ydbio.2006.05.037 16925988

[B30] Ransick A. Davidson E. H. (2012). Cis-regulatory logic driving glial cells missing: self-sustaining circuitry in later embryogenesis. Dev. Biol. 364, 259–267. 10.1016/j.ydbio.2012.02.003 22509525 PMC3561781

[B31] Runcie D. E. Garfield D. A. Babbitt C. C. Wygoda J. A. Mukherjee S. Wray G. A. (2012). Genetics of gene expression responses to temperature stress in a sea urchin gene network. Mol. Ecol. 21, 4547–4562. 10.1111/j.1365-294x.2012.05717.x 22856327 PMC3866972

[B32] Saudemont A. Haillot E. Mekpoh F. Bessodes N. Quirin M. Lapraz F.. (2010). Ancestral regulatory circuits governing ectoderm patterning downstream of Nodal and BMP2/4 revealed by gene regulatory network analysis in an echinoderm. PLoS Genet. 6:e1001259. 10.1371/journal.pgen.1001259 21203442 PMC3009687

[B33] Shen-Orr S. S. Milo R. Mangan S. Alon U. (2002). Network motifs in the transcriptional regulation network of *Escherichia coli*. Nat. Genet. 31, 64–68. 10.1038/ng881 11967538

[B34] Shoval O. Alon U. (2010). SnapShot: network motifs. Cell 143:326–e1. 10.1016/j.cell.2010.09.050 20946989

[B35] Smith J. Kraemer E. Liu H. Theodoris C. Davidson E. (2008). A spatially dynamic cohort of regulatory genes in the endomesodermal gene network of the sea urchin embryo. Dev. Biol. 313, 863–875. 10.1016/j.ydbio.2007.10.042 18061160 PMC3640430

[B36] Smith J. Theodoris C. Davidson E. H. (2007). A gene regulatory network subcircuit drives a dynamic pattern of gene expression. Science 318, 794–797. 10.1126/science.1146524 17975065

